# Serotonin transporter binding is increased in Tourette syndrome with Obsessive Compulsive Disorder

**DOI:** 10.1038/s41598-018-37710-4

**Published:** 2019-01-30

**Authors:** K. R. Müller-Vahl, N. Szejko, F. Wilke, E. Jakubovski, L. Geworski, F. Bengel, G. Berding

**Affiliations:** 1 Clinic of Psychiatry, Socialpsychiatry and Psychotherapy, Hannover Medical School, Hannover, Germany; 2 Department of Neurology, Medical University of Warsaw, Warsaw, Poland; 3 Department of Bioethics, Medical University of Warsaw, Warsaw, Poland; 4 Department of Medical Physics and Radiation Protection, Hannover, Germany; 5 Department of Nuclear Medicine, Hannover Medical School, Hannover, Germany

## Abstract

While the importance of the serotonergic system in obsessive compulsive disorder (OCD) is well established, its role in Tourette syndrome (TS) is uncertain. Particularly in TS patients with comorbid OCD (TS + OCD), decreased serotonin transporter (SERT) binding has been suggested. Here, we investigated for the first time SERT binding in TS patients with and without OCD (TS − OCD) compared to both healthy controls (HC) and OCD patients as well as the influence of escitalopram using the potent SERT imaging ligand [^123^I]2-((2-((dimethylamino)methyl)phenyl)thio)-5-iodophenylamine ([^123^I]ADAM) and single-photon emission tomography (SPECT). We included 33 adult subjects (10 HC, 10 TS − OCD, 8 TS + OCD and 5 OCD). In patients with OCD and TS + OCD [^123^I]ADAM SPECT was repeated after 12–16 weeks treatment with escitalopram. SERT binding was normal in patients with OCD and TS − OCD, but significantly increased (p < 0.05) in those with TS + OCD, particularly in caudate and midbrain compared to both HC and TS − OCD. Treatment with escitalopram resulted in a significant overall reduction in SERT binding (range, 19 to 79%, p values between 0.0409 and <0.0001) without any correlation with clinical improvement. Our results provide further evidence that alterations in the serotonergic system in TS are related to comorbid OCD and do not represent the primary cause of the disease.

## Introduction

Gilles de la Tourette syndrome (TS) is characterized by multiple motor and at least one vocal tic throughout a period of more than 1 year. The onset is before age of 18. Although not included in the diagnostic criteria for TS, it is well known that the majority of patients, in addition, suffer from one or even more psychiatric comorbidities such as attention deficit/hyperactivity disorder (ADHD), obsessive compulsive behavior (OCB) or disorder (OCD), depression, and anxiety disorder. It is thought that TS is caused by alterations in the cortico-striato-thalamo-cortical (CSTC) circuitry. Several different neurotransmitter systems have been suggested to be involved in the neurobiology of TS, but strongest evidence supports a “dopaminergic hypothesis” and, more specifically, an alteration of the tonic-phasic dopamine release system. Since numerous other neurotransmitters are involved in the CSTC pathways including serotonin, endocannabinoids, glutamine, gamma-aminobutyric acid (GABA), acetylcholine, norepinephrine, and opiates, each of these has already been proposed as the underlying pathological factor in TS.

In contrast, in pure OCD it is thought that alterations within the central serotonergic system are the primary cause of the disease. This assumption is mainly supported by the well-known therapeutic effects of (selective) serotonin reuptake inhibitors ((S)SRIs). Similarly, a “serotonin hypothesis“ has also been suggested in depression and anxiety.

In TS, only three neuroimaging studies aimed to investigate the availability of serotonin transporters (SERT) using either [^123^I]ß-carbomethoxy-3ß-(4-iodophenyl)tropane ([^123^I]ß-CIT) and single-photon emission tomography (SPECT)^1,2^ or [^11^C]McN5652 and positron emission tomography (PET)^3^. Although, in all three studies only a small number of adult patients have been included (n = 10^1^, n = 11^3^, and n = 12^2^), the authors consistently reported about reduction of SERT availability in different brain areas including midbrain, caudate, and putamen, respectively. In addition, a negative correlation was found between the severity of vocal tics and [^123^I]ß-CIT binding to SERT in the midbrain and thalamus^1^. Treatment with SSRI resulted in a further reduction of SERT availability^2^. However, it is still unclear, whether these alterations in SERT availability are related to tics or to comorbid OCB/OCD as suggested by some of the studies^2,3^.

In patients suffering from pure OCD, most studies demonstrated reduced brain SERT binding capacity in different brain regions including midbrain, brainstem, thalamus, hypothalamus, limbic and paralimbic brain areas, nucleus accumbens, striatal regions, and insular cortex^4–10^. However, in some studies normal^11,12^ or even elevated^7,13^ binding to SERT has been detected. In addition, a negative correlation between severity of OCD and SERT availability was found^8,9,14^. Interestingly, more recent data provided evidence that SERT availability is only reduced in late, but not in early onset OCD suggesting that distinct subtypes of OCD may differ not only phenomenologically, but also etiologically^7^.

Comparable to preliminary results in patients with TS plus comorbid OCD^2^, patients with pure OCD also showed significantly reduced brain SERT availability in the thalamus–hypothalamus after treatment with (S)SRI^14,15^. Since response to treatment was better in patients with higher pretreatment SERT availability, SERT binding capacity has been suggested as a predictor for positive treatment response using SSRI^14,15^.

It is worth mentioning that altered SERT binding has also been found in other psychiatric disorders than OCD and TS including depression and anxiety disorders. According to a recent meta-analysis, SERT availability is reduced in patients with major depression^16^. In contrast, in patients with anxiety disorders, data are less consistent showing normal^17^ and increased^18^ SERT binding.

This study was designed to further investigate SERT availably in patients with TS compared to healthy controls. In addition, we were interested in further exploring the influence of comorbid OCD, depression, and anxiety on SERT binding capacity in this group of patients and to investigate whether SERT binding in patients with TS plus OCD differs from those with pure OCD. Another aim of this study was to examine the influence of treatment with escitalopram on SERT binding and whether clinical improvement is related to changes in SERT binding. With respect to [I^123^ADAM] SPECT methodology, we aimed to elaborate differences in analyzing with full bio-kinetic modelling compared to simple uptake ratio method, particularly for the assessment of receptor occupancy following SSRI treatment.

In this study, we decided to use the superior SERT imaging agent [^123^I]2-((2-((dimethylamino)methyl)phenyl)thio)-5-iodophenylamine ([^123^I]ADAM) instead of [^123^I]ß-CIT, since [^123^I]ADAM has not only an extremely high binding affinity toward SERT (K(i) = 0.013 nM), but also shows more than 1,000-fold selectivity for SERT over other monoamine transporters. It has an excellent brain stem uptake and consistently displays the highest uptake in the hypothalamus^19^. To the best of our knowledge, until today [^123^I]ADAM and SPECT have not been used to assess SERT binding in patients suffering from both TS and OCD.

## Results

### Clinical findings

All in all, 33 subjects (n = 10 healthy controls, n = 10 patients with TS − OCD, n = 8 with TS + OCD, and n = 5 with pure OCD) were included in this study. The target number of 40 subjects could not be reached, because in 2011 the Federal Institute for Drugs and Medical Devices (Bundesinstitut für Arzneimittel und Medizinprodukte, BfArM) published a red-hand-letter (“Rote-Hand-Brief”) for escitalopram, because of concerns of dose-related prolongation of the QT-interval. Therefore, recruitment had to be stopped at that time.

All 5 patients with pure OCD suffered from moderate to severe OCD as defined according to the Yale-Brown Obsessive Compulsive Scale (Y-BOCS) (≥10 for obsessions or compulsions only and ≥16 for combined obsessions and compulsions). Two patients had early-onset OCD (before age 17), and three late-onset OCD. Three patients with OCD suffered from both obsessions and compulsions (Y-BOCS: range, 16–22) and 2 from compulsions only (Y-BOCS: 10 and 18, respectively). In the TS + OCD group, 7/8 patients suffered from severe OCD; 6 patients had both obsessions and compulsions (Y-BOCS: range, 15–37) and 2 compulsions only (Y-BOCS: 12 and 13, respectively). The diagnosis of ADHD was made in 2 patients with TS + OCD (detailed results of the Conners’ Adult ADHD Rating Scale (CAARS) and the Wender Utah Rating Scale short version (WURS-K) not shown), the diagnoses of depression (according to the Beck Depression Inventory, BDI) in 1 patient with OCD, 1 with TS − OCD, and 3 with TS + OCD, and anxiety (according to the State-Trait Anxiety Inventory, STAI) in 1 patient with OCD, 4 with TS − OCD, and 9 with TS + OCD. Multiple choice vocabulary test (Mehrfachwahl-Wortschatztest, MWT-B) demonstrated normal intelligence in all subjects. However, mean scores of MWT-B were significantly lower in patients with TS + OCD (27.25 ± 5.75) compared to those with TS − OCD (31.18 ± 2.52, p = 0.05) (detailed results of MWT-B not shown). None of the healthy control subjects suffered from tics, OCB, depression, and ADHD, but 4 from anxiety according to above-mentioned assessments. One patient with TS + OCD received methylphenidate for the treatment of comorbid ADHD; another patient with TS + OCD received treatment for tics with tetrabenazine and pimozide. All other patients were drug-free. Further clinical details are summarized in Table 1.

**Table 1:** Demographic and clinical data at baseline and - in patients with OCD - after treatment with escitalopram.

	Controls	TS − OCD	TS + OCD			Pure OCD		
N	10	10	8			5		
Male/female [n]	8/2	10/0	7/1			3/2		
Mean age ± SD (range) [years]	40 ± 16 (19–61)	40 ± 14 (21–64)	33 ± 10 (19–45)			47 ± 4 (41–53)		
ADHD [n/%]	0/0%	0/0%	2/25%			0/0%		
Depression [n/%]	0/0%	1/10%	3/37.5%			1/20%		
Anxiety disorder [n/%]	4/40%	4/40%	8/100%			1/20%		
			before	after	p	before	after	p
			treatment with escitalopram			treatment with escitalopram		
YGTSS	0	16 ± 4	23 ± 10	23 ± 10	0.66	0	0	
Y-BOCS	0	0	21 ± 8	18 ± 5	0.22	17 ± 4	16 ± 5	0.85
BDI	2.2 ± 3.2	6 ± 6	13 ± 5	8 ± 6	0.04	13 ± 6	12 ± 14	0.48
STAI-X1	33.9 ± 8.4	37.8 ± 8.0	44 ± 11.4	44.1 ± 14.7	0.24	46.7 ± 10.8	47.2 ± 10.3	0.58
SCL-90-GSI	0.2 ± 0.1	1.3 ± 0.3	1.7 ± 0.5	1.5 ± 0.4	0.34	1.7 ± 0.5	1.70 ± 0.7	0.32

YGTSS = Yale Global Tic Severity Scale (range, 0–50), Y-BOCS = Yale-Brown Obsessive Compulsive Scale (range, 0–40), BDI = Beck’s Depression Inventory (range, 0–63), STAI-X1 = State-Trait Anxiety Inventory (X1 = state, range, 20–80), SCL-90-GSI = Symptom Check List 90 Revised, Global Severity Index, TS − OCD = Tourette syndrome without obsessive compulsive behavior, TS + OCD = Tourette syndrome with obsessive compulsive disorder, OCD = obsessive compulsive disorder.

All patients with TS + OCD (n = 8) and pure OCD (n = 5) received treatment with escitalopram (mean dosage 25.77 ± 8.28, range, 5–30 mg) for OCD. In one patient starting dose of 10 mg had to be reduced to 5 mg/day due to reduced libido. In patients with TS + OCD, treatment with escitalopram had no effects on tics (according to YGTSS-TTS, p = 0.6682,), anxiety (according to STAI-X1, state-anxiety, p = 0.2453), or general psychological symptomatology and distress (according to the general symptomatic index (GSI) of the SCL-90-R, p = 0.3467), but resulted in a non-significant improvement of OCD (according to Y-BOCS) and significantly improved depression (according to BDI, p = 0.0417). In patients with pure OCD, no treatment effects were seen in any of the assessments used (for details see Table 1). During treatment with escitalopram, 7/13 patients reported adverse events (AEs) (multiple answers possible) including reduced libido (n = 3), reduced sexual potency (n = 2), sedation, restlessness, problems with concentration, sleeping problems, weight gain, and mild gastrointestinal problems (each, n = 1). No serious AEs occurred and in no case any intervention was necessary because of AEs. None of the patients stopped medication prematurely (before second SPECT scan), but some patients used only low dosages.

### SERT binding at baseline

In patients with TS − OCD, SERT binding was significantly increased in pons compared to healthy controls (p = 0.0335). This difference, however, was only significant when using one procedure of quantification (Logan fitted), but not with others (Logan fixed, ratio). In contrast, in patients with TS + OCD (compared to healthy controls), SERT binding was significantly increased in multiple brain areas including caudate (p = 0.0284), hypothalamus (p = 0.0227), and midbrain (p = 0.0191). In addition, binding was increased compared to patients with TS − OCD in caudate (p = 0.0216), midbrain (p = 0.0479), and thalamus (p = 0.0491). Most robust results could be detected in caudate (TS + OCD vs. both TS − OCD (p = 0.0216) and healthy controls (p = 0.0284)) and midbrain (TS + OCD vs. healthy controls, p = 0.0191), since significant differences could be detected using different procedures of quantification. In patients with pure OCD, no differences could be detected compared to any other group (TS − OCD, TS + OCD, healthy controls). There was no difference between patients with early and late onset OCD (detailed comparisons of all groups are shown in Table 2).

**Table 2:** Comparison of SERT binding (at baseline) between different patient groups (TS − OCD, TS + OCD, OCD) and healthy controls (HC), respectively, using different procedures of quantification.

Group comparison	Brain region	Analysis method	SERT binding (Means, *p < 0.05)
TS − OCD vs. HC	Pons	Logan fitted	1.46 vs. 1.36* (p = 0.0335)
TS + OCD vs. HC	Caudate	Logan fixed	1.59 vs. 1.43* (p = 0.0284)
	Caudate	Logan fitted	1.61 vs. 1.46* (p = 0.0377)
	Hypothalamus	Ratio	2.38 vs. 2.13* (p = 0.0227)
	Midbrain	Logan fixed	1.95 vs. 1.79* (p = 0.0191)
	Midbrain	Logan fitted	1.99 vs. 1.83* (p = 0.0127)
TS + OCD vs. TS − OCD	Caudate	Logan fixed	1.59 vs. 1.40* (p = 0.0348)
	Caudate	Logan fitted	1.61 vs. 1.36* (p = 0.0216)
	Midbrain	Logan fitted	1.99 vs. 1.81* (p = 0.0479)
	Thalamus	Logan fixed	1.94 vs. 1.72* (p = 0.0491)
OCD vs. HC, TS + OCD, and TS − OCD	all	all	n.s.

SERT = serotonin transporter, TS − OCD = Tourette syndrome without obsessive compulsive behavior, TS + OCD = Tourette syndrome with obsessive compulsive disorder, OCD = obsessive compulsive disorder, HC = healthy controls.

### Correlations between SERT binding at baseline and clinical data

We found no correlation between baseline SERT binding and OCD (according to Y-BOCS), neither in patients with pure OCD, nor in those with TS + OCD. In addition, the type of OCD (obsessions, compulsions or both) had no influence on this result. There was also no correlation between SERT availability and depression (according to BDI) and anxiety (according to STAI-X1 and –X2), respectively, in the patients’ group (TS, TS − OCD, and TS + OCD, n = 23). However, in patients with TS (with and without OCD, n = 18), tic severity (according to YGTSS-TTS) was negatively correlated with SERT binding in pons (Logan fixed: r-sq = 0.2320, p = 0.0429, Logan fitted: r-sq = 0.2623, p = 0.0298) – but no other brain area - suggesting that SERT binding is higher in less severely affected patients.

### Influence of escitalopram on SERT binding

**Figure 1:**
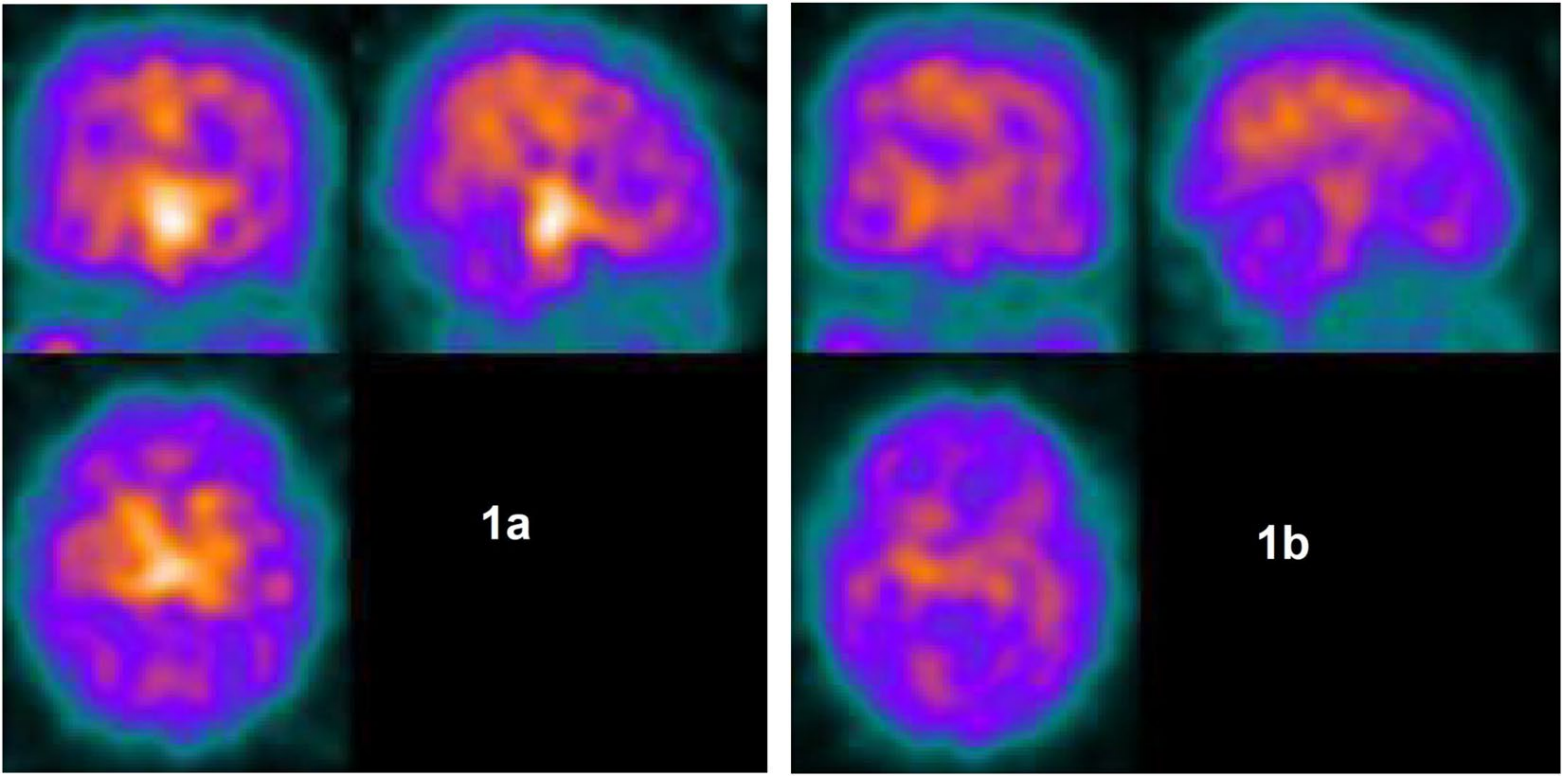
Example of [123I]ADAM uptake in a patient with Tourette syndrome and obsessive compulsive disorder (TS + OCD). In each figure, three perpendicular cross-sections before (**a**) and after (**b**) treatment with escitalopram are displayed illustrating a significant reduction of serotonin transporter (SERT) binding after treatment. Prominent displacement of tracer uptake in the brainstem is evident.

**Figure 2:**
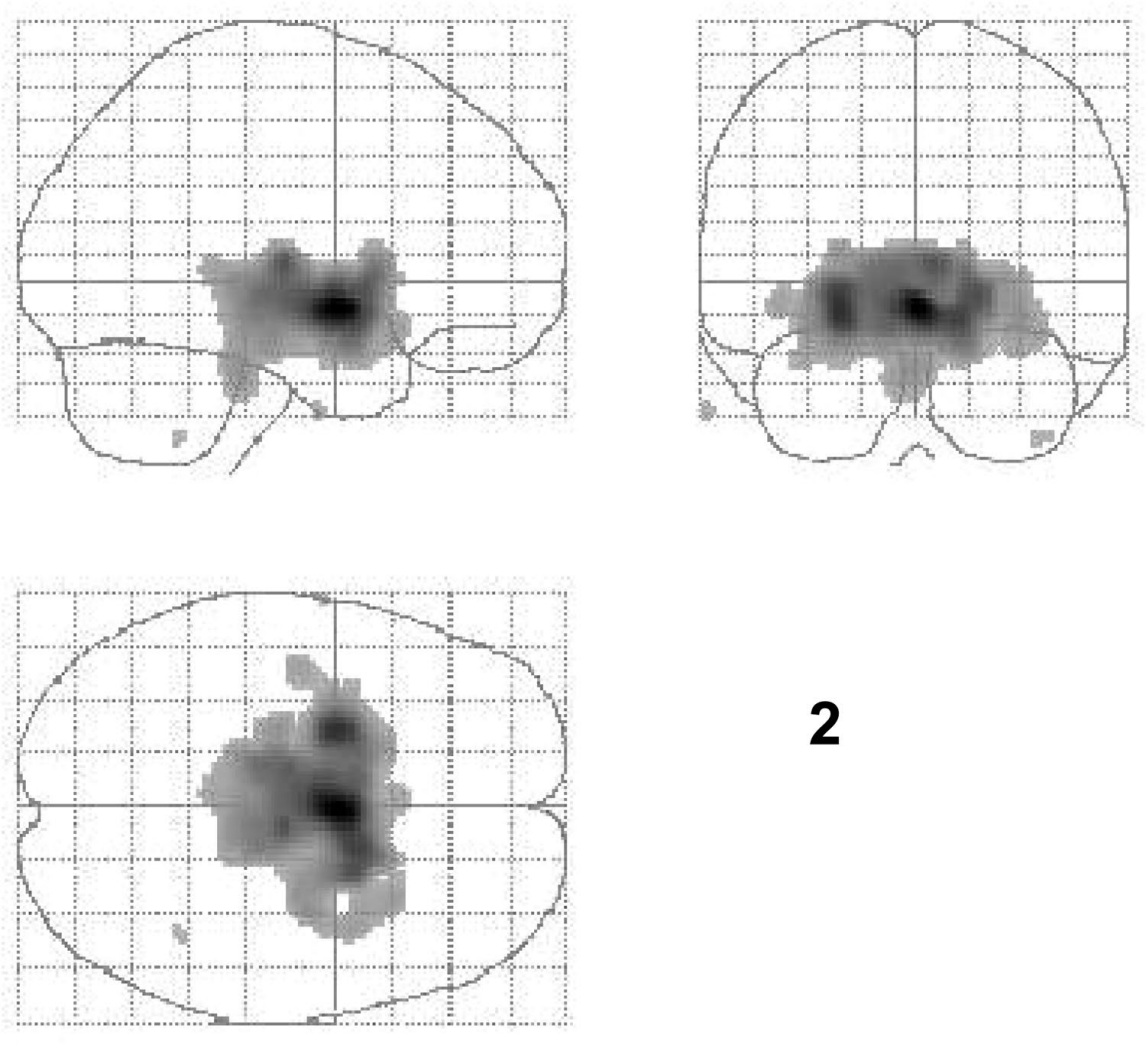
Results of SPM analysis (glass brain display of SPM-analysis, threshold for statistical inferences: uncorrected p < 0.001) in patients with Tourette syndrome plus obsessive compulsive disorder (TS + OCD, n = 8) are shown. Regions with significantly reduced serotonin transporter (SERT) binding ([123I]ADAM uptake) after treatment with escitalopram are illustrated as compared to the untreated state. Extended reductions of SERT binding are visible in brainstem, hypothalamus, and striatum.

Treatment with escitalopram in patients with TS + OCD and pure OCD (n = 13) resulted in a significant reduction of SERT binding in all investigated brain areas including caudate, putamen, thalamus, hypothalamus, midbrain, pons, and mesial temporal cortex (p values between 0.0409 and <0.0001 depending on the brain region) with differences ranging from 19 to 78% (for further details see Table 3, and Figs 1 and 2). Thus, treatment with escitalopram resulted in a much larger difference in SERT binding compared to the differences detected between untreated patients (with TS + OCD and OCD) and healthy controls at baseline (range, 8 to 11%, for further details see Table 2).

**Table 3:** SERT occupancy (=percentage of decrease in specific over non-specific binding due to treatment) in different brain regions after treatment with escitalopram in patients with TS + OCD and pure OCD (n = 13) depending on quantification procedure.

Brain region	Analysis method	SERT occupancy [%]	T-test [p]
Caudatus	Ratio	36	0.0005
	Logan fixed	32	0.0016
	Logan fitted	35	0.0007
Hypothalamus	Ratio	50	0.0011
	Logan fixed	73	<0.0001
	Logan fitted	79	<0.0001
Midbrain	Ratio	57	<0.0001
	Logan fixed	53	<0.0001
	Logan fitted	56	<0.0001
Mesial temporal cortex	Ratio	19	0.0179
	Logan fixed	29	0.0004
	Logan fitted	31	0.0004
Pons	Ratio	25	0.0409
	Logan fixed	49	0.0003
	Logan fitted	48	0.0009
Putamen	Ratio	30	0.001
	Logan fixed	32	<0.0001
	Logan fitted	34	<0.0001
Thalamus	Ratio	38	0.0009
	Logan fixed	30	0.0005
	Logan fitted	33	0.0007

SERT = serotonin transporter, TS + OCD = Tourette syndrome with obsessive compulsive disorder, OCD = obsessive compulsive disorder.

### Comparison of bio-kinetic modelling and ratio method

In all investigated brain areas, we found relevant differences between results obtained from bio-kinetic modelling compared to those based on ratio method. Throughout, values of specific SERT binding assessed with the ratio method were significantly higher compared to those obtained from bio-kinetic modelling. In contrast, differences between results obtained using Logan fixed compared to Logan fitted modelling procedure were only minimal. Significant differences were found only in 2 (hypothalamus, mesial temporal cortex) out of 7 brain areas investigated (see Table 4). Bio-kinetic modelling revealed a higher level of occupancy compared to the ratio method (see Table 3). Consequently, in most cases, the levels of significance in differences between SERT binding at baseline and after treatment were higher for values obtained from bio-kinetic modeling compared to ratio method (see Table 3). Furthermore, differences between different patients’ groups were mainly detected by using bio-kinetic modelling (see Table 2). In summary, most of our results based on dynamic data acquisition and analysis employing bio-kinetic modelling. Simplification by relying only on acquisition of one late frame and analysis by ratio method seems to be insufficient.

**Table 4:** Serotonin transporter (SERT) binding in different brain areas determined using ratio method or bio-kinetic modelling (with Logan plot analysis with fixed or fitted k2, respectively).

	Ratio	Logan (k2 fixed)	Logan (k2 fitted)
Caudate	1,69^*^	1,43	1,43
Hypothalmus	2,05^*^	1,64^#^	1,57^#^
Midbrain	2,10^*^	1,74	1,74
MTC	1,69^*^	1,45^#^	1,47^#^
Pons	1,63^*^	1,35	1,36
Putamen	1,85^*^	1,67	1,65
Thalamus	1,95^*^	1,76	1,76

^*^Significantly higher values obtained with the ratio method compared to both Logan procedures are indicated with a star (significant differences, t-test < 0.0001).

^#^Significant differences between values obtained with both Logan procedures are marked with a hashtag (significant differences, t-test < 0.0001).

MTC = mesial temporal cortex.

## Discussion

This is the very first study using SPECT and the highly affine and specific radioligand [^123^I]ADAM to investigate SERT binding in patients with TS and OCD compared to healthy controls. In TS, so far only less potent radioligands for SPECT ([^123^I]ß-CIT)^1,2^ or PET ([^11^C]McN5652) have been used to investigate SERT binding^3^. This is also the first study that directly compares SERT binding in TS patients with (TS + OCD) and without (TS − OCD) comorbid OCD. The main results of this study are (i) SERT binding is significantly increased in patients with TS + OCD in caudate and midbrain compared to both, healthy controls and patients with TS − OCD and (ii) in patients with OCD (with and without TS), treatment with escitalopram results in a significant overall reduction in SERT binding. Although this is the largest study investigating SERT binding in patients with TS, the sample size is still relatively small, which limits the interpretability of the results. We were unable to detect any changes of SERT binding in patients with pure OCD compared to both patients with TS (with and without OCD) and healthy controls. However, in this group only 5 patients could be included before recruitment had to be stopped prematurely, because the German authorities (BfArM) had published a so called “red-hand letter”, restricting the use of escitalopram. Moreover, escitalopram was not well tolerated in all patients and therefore, some patients received only low dosages, because up-titration was not possible. In these patients, we decided to continue medication, although it is well known that in OCD efficacy of low doses is insufficient. Not surprisingly, in this small group of patients we failed to detect differences in SERT binding between patients with early-onset compared to late-onset OCD. Thus, further interpretation of the data is difficult. However, from available data it can be speculated that the underlying pathology in pure OCD is different from the pathology of OCD in the context of TS.

We believe that our finding of increased SERT binding in pons (but not in any other brain region) in patients with TS without OCD (TS − OCD) compared to healthy controls should be interpreted with caution for several reasons: (i) it is well known that considerably higher uptake values can be detected in brain stem and hypothalamus as compared to the pons^20^ resulting in less certainty of values determined for the pons; (ii) differences could be detected only when using Logan fitted analysis (but not with Logan fixed and ratio methods) as quantification procedure further supporting the questionable significance of this result. For a reliable result, one would also expect a significant result, when using Logan fixed method; (iii) when comparing SERT binding in pons in patients with TS − OCD and TS + OCD, no differences could be found. In contrast, several other regions exhibited increased binding in TS + OCD compared to TS − OCD; and (iv) so far, changes of SERT binding in pons have been rarely reported in the literature. Taking together, we believe that from our data it is suggested that in patients with TS without comorbid OCD (TS − OCD), no relevant changes in the serotonergic system are present.

In patients with TS + OCD, SERT binding was significantly increased in multiple brain areas. However, we believe that only differences detected in caudate and midbrain should be interpreted as relevant results due to the following reasons: (i) only in these two brain regions differences were significant compared to both, patients with TS − OCD and healthy controls; (ii) a significant difference in the thalamus could be found only compared to patients with TS − OCD (but not compared to healthy controls) and only when using one (Logan fixed) out of three quantification procedures; (iii) comparably, a significant difference in the hypothalamus could be detected only compared to healthy controls (but not to patients with TS − OCD) and only with ratio method (but not with bio-kinetic modelling). This result should therefore be interpreted with caution, since hypothalamus is among those brain areas that generally demonstrates high uptake values and ratio methods overestimates SERT binding.

For many years, there has been an intense and controversial discussion about the involvement of the serotonergic system in the pathology of TS, however, the findings are contradictory: While in some studies alterations in the serotonergic system could be detected – for example reduced levels of 5-hydroxyindoleacetic acid (5-HIAA) in cerebrospinal fluid^21,22^ and basal ganglia^23^, and reduced plasma tryptophan and blood serotonin levels^24^ - others failed to demonstrate alterations and found normal levels of 5-HIAA in the cerebral cortex^25^ and normal density of cerebral 5-hydroxytryptamine (HT)-1A receptors^26^. Furthermore, in genetic studies investigating genes^27^ and single nucleotide polymorphisms (SNPs) in genes^28^ belonging to the serotonergic system results were also inconsistent. Thus, it can be speculated that alterations in the serotonergic system are present in some, but not all patients with TS. Our results, therefore, lend further support to the hypothesis that changes in SERT binding are restricted to those patients with TS with comorbid OCD (TS + OCD), but are not present in patients with pure TS (without OCD) as suggested earlier^2^. Accordingly, changes in the serotonergic system seem to be unrelated to the pathophysiology of tics.

However, in contrast to all recent imaging studies in TS^1–3^, in this study SERT binding was *increased* in patients with TS + OCD in caudate and midbrain (and possibly further brain areas), while other investigators reported about significantly *decreased* SERT binding potentials in the midbrain^2,3^, caudate, and putamen^2,3^ or no change^1^ compared to healthy controls. We found in none of the investigated brain regions decreased SERT binding potentials compared to healthy controls. This discrepancy might be explained by methodological differences such as different imaging techniques (SPECT vs. PET), different radio-ligands ([^123^I]ADAM vs. [123I]ß-CIT), and different procedures of data analysis used. Moreover, differences in patients’ age, tic severity, comorbidities, and medication cannot be excluded as relevant factors, since in all available studies only small sample sizes (between 10 to 12 patients) have been investigated. Remarkably, our finding of increased activity in the serotonergic system in patients with TS + OCD is in agreement with results from Haugbøl *et al*.^29^, who reported about globally increased 5-HT2A receptor binding using PET-[^18^F]altanserin. Interestingly, in this study 7/12 patients also suffered from comorbid OCD. However, no correlations with clinical data could be detected. The authors speculated that increased 5-HT2A binding could be caused by a reduction in synaptic serotonin levels. In line with this assumption, decreased cerebral serotonin levels have already been found in TS^30^. It is noteworthy that in patients with pure OCD, it has been suggested that SERT availability is *reduced* only in patients with late-onset OCD^7^, but *increased* in those with early-onset OCD in the putamen^31^ and midbrain-pons^13^, respectively. It, therefore, can be speculated that early-onset OCD is not only a distinct subtype of OCD^7^, but may etiologically be closely related to OCD in patients with TS, since in both groups SERT binding is increased in comparable brain regions. Finally, our data are in line with results from an animal model for TS (Wilstar rats) demonstrating a higher expression of SERT in the striatum compared to controls^32^. The authors suggest that increased SERT might be the consequence of reduced serotonin levels, since the concentration of serotonin in the neuron is regulated by SERT via reuptake from the synaptic cleft. Comparable to our earlier study in TS^2^ and in agreement with several other studies^33^, treatment with an SSRI resulted in a significant overall reduction of SERT binding.

In patients with TS + OCD, treatment with escitalopram resulted in a significant improvement of depression, but failed to improve OCD and anxiety. As expected, tics did not improve during treatment with escitalopram^34^. In patients with pure OCD, no treatment effects were seen, which might be explained by (i) the small sample size, (ii) the fact that most patients suffered from severe OCD, (iii) dosage of escitalopram was low in some patients, and (iv) in most patients other treatment strategies (including different SSRI) already failed to improve symptoms.

We employed [^123^I]ADAM, a potent SERT imaging ligand. As demonstrated earlier^35^, semi-quantification of static [^123^I]ADAM images using the ratio method resulted in an overestimation of SERT binding, when compared to quantitative analysis of the entire dynamic SPECT studies. Therefore, we recommend bio-kinetic modeling of dynamic data as the method of choice for the evaluation of [^123^I]ADAM SPECT studies.

Compared to recent studies investigating SERT binding potentials in patients with TS, this study has the following strengths: (i) inclusion of a larger number of patients with TS, (ii) a priori differentiation between patients with and without comorbid OCD, (iii) all but one patient with TS + OCD suffered from severe OCD, (iv) no influence of medication, (v) use of a sophisticated imaging methodology using an ADAM template complying with the MNI stereotaxic space based on MRI and SPECT data from healthy controls including fiducial markers, and (vi) use of the highly affine and selective radioligand [^123^I]ADAM, which is superior to [^123^I]ß-CIT.

The following limitations of the study have to be addressed: (i) although this is the largest study investigating SERT binding in patients with TS, the number of patients included is still relatively small and this limits the interpretation of our findings; (ii) since recruitment had to be stopped prematurely, we are unable to make any conclusive statement regarding SERT binding in patients with pure OCD (because only 5 patients could be included); (iii) although the second SPECT scan was performed after a quite long period of 12–16 weeks after initiation of medication with escitalopram, one might speculate that a longer treatment period might have resulted in discrepant findings and further reduction of SERT binding; (iv) although we found no correlation between SERT availability and depression and anxiety, respectively, we cannot entirely exclude an influence of these comorbidities on SERT binding, since it has been demonstrated that SERT availability is reduced in patients with major depression (without TS)^16^, while data in patients with anxiety disorders are inconsistent^17,18^; (v) another limitation of the present study is the spatial resolution of the employed SPECT system in comparison to the resolution that would have been available from a PET system. The limitation in resolution might induce (in small imaged objects) partial volume effects, i.e. an underestimation of the signal (contrast) to be measured. Specifically, the spatial resolution of the SPECT system in terms of FWHM (full width at half maximum) is 9–10 mm^36^, but only 3–4 mm using modern PET systems^31^. The diameters of the objects measured in this study – specifically e.g. the caudate nucleus and the pons - are about 9–12 mm^37^ and 25–29 mm^38^, respectively. In emission tomography, the recovery of the signal from an object with the diameter around 1-times the FWHM (here: SPECT & the caudate nucleus) can drop down to 20%^39^. Accordingly, in this SPECT study, we considerably underestimated the signal in some of the evaluated brain regions. This could have been prevented by using PET, because of a 90% signal recovery for the caudate nucleus with a diameter of about 3-times the FWHM^40^. However, there is general agreement that signals obtained from SPECT tracers binding to basal ganglia structures lead to meaningful results^41^. Specifically for the SPECT ligand ^123^I-ADAM, it could be demonstrated that SERT binding in basal ganglia and midbrain structures can be measured with acceptable accuracy and reproducibility^42,43^.

In summary, we found *increased* SERT binding in patients with TS and comorbid OCD (TS + OCD) in caudate and midbrain compared to both, healthy controls and patients with TS − OCD. In line with recent findings, changes in SERT binding could be detected only in patients with TS with comorbid OCD. Thus, our data further support the hypothesis that alterations in the serotonergic system in patients with TS are related to comorbid OCD, but do not represent the primary cause of the disease. Since we found no changes in SERT binding in a small number of patients with pure OCD, one might speculate that underlying pathology is different compared to OCD in patients with TS. However, it can be speculated that OCD in TS is etiologically related to early-onset OCD, since in both groups SERT binding is increased.

## Methods

### Subjects

In this study, we aimed to include 40 subjects, namely 10 patients with TS without comorbid OCD (TS − OCD), 10 with TS plus comorbid OCD (TS + OCD), 10 with pure OCD, and 10 healthy controls. All assessments took place between 11/2006 and 08/2010. Patients were recruited from the Tourette outpatient clinic at the Hannover Medical School and the German Tourette advocacy group (Tourette Gesellschaft Deutschland e.V.). For all patients, the DSM-IV-TR diagnoses of TS and OCD were confirmed by one of the authors (KMV). The following exclusion criteria were defined: (1) age < 18, (2) secondary tic disorder (for patients with TS), (3) tics (for patients with pure OCD), (4) additional severe medical, neurological, or psychiatric diseases such of psychosis, epilepsy, addiction, and clear mental retardation, (5) treatment with an (S)SRI or any other drug that influences the central serotonergic system 6 months before entering the study, (6) during the study no treatment with any substance influencing the central serotonergic system was allowed beside the study medication; in addition, initiation of behavioral therapy for OCD or tics was not allowed, and (7) pregnancy and breast feeding. Common psychiatric comorbidities of TS (such as ADHD, OCD, depression, anxiety) as well as stable medication for tics and comorbidities (not influencing the serotonergic system) were no exclusion criteria. Patients received reimbursement for travel costs. In addition, a reference group of sex- and age-matched healthy, drug-free controls (≥18 years) was enrolled. They were matched to both patients’ groups (TS and OCD). All control subjects received monetary compensation for participating in the study. Demographic data are summarized in Table 1. All subjects gave written informed consent before entering the study. The study was approved by the ethics committee of the Hannover Medical School (no. 3792), the Federal Office for Radiation Protection (BfS, no. Z 5-22461/2-2005-039) and the Federal Institute for Drugs and Medical Devices (BfArM, EudraCT no. 2005-001411-22). All methods were performed in accordance with the relevant guidelines and regulations.

### Clinical assessments

For clinical characterization of the patients’ group we used several different well established assessments: (1) Yale Global Tic Severity Scale (YGTSS)^44^ including YGTSS – total tic score (TTS) and YGTSS – global score (GS) (=YGTSS-TTS + impairment score) to assess tic severity, (2) Yale-Brown Obsessive Compulsive Scale (Y-BOCS) for the assessment of obsessive-compulsive symptoms^45^, (3) Beck’s Depression Inventory (BDI) to measure depressive symptoms^46^, (4) State-Trait-Anxiety Inventory (STAI) to assess anxiety (X1 = state anxiety, X2 = trait anxiety)^47^, (5) Conners’ Adult ADHD Rating Scale (CAARS)^48^ as well as (6) Wender Utah Rating Scale short version (WURS-K)^49^, which was used in combination with a clinical interview to make the diagnosis of current ADHD, (7) Symptom Checklist 90-R (SCL-90-R) to measure the following psychological symptoms: somatization, OCB, interpersonal sensitivity, depression, anxiety, anger-hostility, phobic anxiety, paranoid ideation, and psychoticism. In addition, a general symptomatic index (GSI), a positive symptom total (PST) and a positive symptom distress index (PSDI) is calculated^50^, and (8) Multiple choice vocabulary test (Mehrfachwahl-Wortschatztest, MWT-B)^51^ was used to measure verbal intelligence. Same assessments were performed in control subjects to exclude psychiatric symptoms. In patients with TS − OCD and normal controls, clinical assessments (and SPECT imaging) were performed only once at baseline. In those with TS + OCD and pure OCD, all clinical assessments (and SPECT imaging) were repeated after treatment with SSRI (for details see below).

### Image data acquisition

All subjects underwent dynamic SPECT imaging using a dual head SPECT camera (EACM variable, Siemens, Erlangen, Germany) starting immediately after injection with 185 MBq I-123-ADAM (MAP Medical, FIN) and continuing up to 5 hours with pauses in between. While patients with TS − OCD and healthy controls were scanned only once (at baseline), all other patients (TS + OCD, pure OCD) were investigated for a second time 12–16 weeks after treatment with escitalopram (for treatment details see below).

For SPECT, the following sequences of frames were used: 15 × 5 min, 31 min pause, 2 × 10 min, 19 min pause, 2 × 10 min, 29 min pause, 3 × 10 min, 37 min pause, 4 × 10 min. Scanning was performed alternately clock and counter-clock wise in order to minimize time of gantry rotation without acquisition. Scans were acquired in continuous rotation mode with a circular orbit. Images were collected with high-resolution collimators: 120 projections, matrix size 128 × 128, pixel size 3 mm. The SPECT image data were reconstructed using filtered back-projection with a Butterworth filter (cut-off 0.4, order 7) including Chang’s attenuation correction (coefficient µ = 0.12).

Additionally, in each subject an isotropic T1-weighted structural magnetic resonance imaging (MRI) data set was acquired to exclude brain pathologies. In all cases, SPECT and MRI were performed at the same day. For later image data analysis during MRI and SPECT, six fiducial markers containing Co-57 were fixed at the patient’s head using adhesive tape.

### Image data analysis

For data analyses, we used a statistical parametric mapping software (SPM2, Wellcome Trust Centre for Neuroimaging, London, UK) and PMOD version 2.8 (PMOD Technologies LTD, Zurich, Switzerland). In order to achieve spatial normalization of SPECT data in the patients’ group, we generated an ADAM SPECT template according to MNI (Montreal Neurologic Institute) space based on MRI and SPECT in healthy controls. All SPECT studies were spatially normalized using this template. For quantification we used the cerebellum as a reference to calculate: specific over non-specific binding ratios (of normalized count densities in static SPECT at 5 h p.i.) and distribution volume ratios (DVR) based on the Logan reference tissue model (employing the dynamic SPECT study up to 5 h p.i.) with fixed k2 = 0.03, as well as individually fitted k2^35^. Furthermore, percentages of decrease in specific over non-specific binding due to treatment (SERT occupancy) were calculated.

Parameters of SERT binding were regionally calculated for volumes of interest (VOI) according to WFU (Wake Forest University) Pick Atlas: (1) hypothalamus, (2) midbrain, (3) pons, (4) caudate, (5) putamen, (6) thalamus, and (7) mesial temporal cortex (MTC). In addition, images were analysed voxel-wise using SPM2.

### Statistical analysis

Statistical analysis has been performed using JMP 10 software (SAS Institute Inc.) with a threshold to assess significance of p < 0.05. Paired t-tests have been used to compare clinical assessments, SERT binding before and after treatment, as well as parameters of SERT binding obtained with different approaches. Unpaired t-tests have been employed to compare different patient groups among each other and with normal controls.

### Treatment with the SSRI escitalopram

After baseline data acquisition (clinical assessment, MRI, and SPECT imaging), all patients suffering from OCD (both patients with TS + OCD and with pure OCD) received oral treatment with escitalopram. Medication was administered once in the morning, starting dose was 10 mg/day. Dose was increased gradually by 10 mg/week, if well tolerated up to a maximum dose of 30 mg/day, followed by a maintenance phase. Follow-up investigations (clinical assessments, MRI, and SPECT imaging) were performed after a treatment period of 12–16 weeks. Thereafter, medication was down-titrated by 10 mg/week or, if well tolerated and effective, continued.

## References

[1] Heinz A (1998). Tourette’s syndrome: [I-123] beta-CIT SPECT correlates of vocal tic severity. Neurology.

[2] Müller-Vahl KR (2005). Serotonin transporter binding in Tourette Syndrome. Neurosci. Lett..

[3] Wong DF (2008). Mechanisms of dopaminergic and serotonergic neurotransmission in Tourette syndrome: clues from an *in vivo* neurochemistry study with PET. Neuropsychopharmacol. Off. Publ. Am. Coll. Neuropsychopharmacol..

[4] Zitterl W (2007). [123I]-??-CIT SPECT imaging shows reduced thalamus-hypothalamus serotonin transporter availability in 24 drug-free obsessive-compulsive checkers. Neuropsychopharmacology.

[5] Hasselbalch SG (2007). Reduced midbrain-pons serotonin transporter binding in patients with obsessive-compulsive disorder. Acta Psychiatr. Scand..

[6] Hesse S (2005). Serotonin and dopamine transporter imaging in patients with obsessive-compulsive disorder. Psychiatry Res. - Neuroimaging.

[7] Hesse S (2011). The serotonin transporter availability in untreated early-onset and late-onset patients with obsessive-compulsive disorder. Int. J. Neuropsychopharmacol..

[8] Reimold M (2007). Reduced availability of serotonin transporters in obsessive-compulsive disorder correlates with symptom severity - A [11C]DASB PET study. J. Neural Transm..

[9] Matsumoto R (2010). Reduced serotonin transporter binding in the insular cortex in patients with obsessive-compulsive disorder: A [11C]DASB PET study. Neuroimage.

[10] Kim E (2016). Altered serotonin transporter binding potential in patients with obsessive-compulsive disorder under escitalopram treatment: [11C]DASB PET study. Psychol. Med..

[11] Simpson HB (2003). Serotonin transporters in obsessive-compulsive disorder: A positron emission tomography study with [11C]McN 5652. Biol. Psychiatry.

[12] Van Der Wee NJ (2004). Enhanced dopamine transporter density in psychotropic-naive patients with obsessive-compulsive disorder shown by [123I]β-CIT SPECT. Am. J. Psychiatry.

[13] Pogarell O (2003). Elevated brain serotonin transporter availability in patients with obsessive-compulsive disorder. Biol. Psychiatry.

[14] Zitterl W (2008). Changes in thalamus-hypothalamus serotonin transporter availability during clomipramine administration in patients with obsessive-compulsive disorder. Neuropsychopharmacology.

[15] Stengler-Wenzke K (2006). Serotonin transporter imaging with [123I]beta-CIT SPECT before and after one year of citalopram treatment of obsessive-compulsive disorder. Neuropsychobiology.

[16] Gryglewski G, Lanzenberger R, Kranz GS, Cumming P (2014). Meta-analysis of molecular imaging of serotonin transporters in major depression. Journal of Cerebral Blood Flow and Metabolism.

[17] Maron E (2004). SPECT imaging of serotonin transporter binding in patients with generalized anxiety disorder. Eur. Arch. Psychiatry Clin. Neurosci..

[18] van der Wee NJ (2008). Increased serotonin and dopamine transporter binding in psychotropic medication-naive patients with generalized social anxiety disorder shown by 123I-beta-(4-iodophenyl)-tropane SPECT. J. Nucl. Med..

[19] Oya S (2000). 2-((2-((Dimethylamino)methyl)phenyl)thio)-5-iodophenylamine (ADAM): An improved serotonin transporter ligand. Nucl. Med. Biol..

[20] Lin K-J (2006). Brain SPECT imaging and whole-body biodistribution with [(123)I]ADAM - a serotonin transporter radiotracer in healthy human subjects. Nucl. Med. Biol..

[21] Butler IJ, Koslow SH, Seifert WE, Caprioli RM, Singer HS (1979). Biogenic amine metabolism in tourette syndrome. Ann. Neurol..

[22] Singer HS, Tune LE, Butler IJ, Zaczek R, Coyle JT (1982). Clinical symptomatology, CSF neurotransmitter metabolites, and serum haloperidol levels in Tourette syndrome. Adv. Neurol..

[23] Anderson GM (1992). Brain Monoamines and Amino Acids in Gilles de la Tourette’s Syndrome: A Preliminary Study of Subcortical Regions. Archives of General Psychiatry.

[24] Comings DE (1990). Blood serotonin and tryptophan in Tourette syndrome. Am. J. Med. Genet..

[25] Singer HS, Hahn IH, Krowiak E, Nelson E, Moran T (1990). Tourette’s syndrome: a neurochemical analysis of postmortem cortical brain tissue. Ann. Neurol..

[26] Minzer K, Lee O, Hong JJ, Singer HS (2004). Increased prefrontal D2 protein in Tourette syndrome: A postmortem analysis of frontal cortex and striatum. J. Neurol. Sci..

[27] Qi, Y., Zheng, Y., Li, Z. & Xiong, L. Progress in genetic studies of tourette’s syndrome. *Brain Sciences* **7** (2017). 10.3390/brainsci7100134 PMC5664061 29053637

[28] Abdulkadir, M. *et al*. Investigation of previously implicated genetic variants in chronic tic disorders: a transmission disequilibrium test approach. *European Archives of Psychiatry and Clinical Neuroscience* 1–16, 10.1007/s00406-017-0808-8 (2017). 10.1007/s00406-017-0808-8 PMC5708161 28555406

[29] Haugbol S (2007). Cerebral 5-HT2A receptor binding is increased in patients with Tourette’s syndrome. Int J Neuropsychopharmacol.

[30] Anderson GM (1992). Postmortem analysis of subcortical monoamines and amino acids in Tourette syndrome. Adv. Neurol..

[31] Lee YS (2014). Performance measurement of PSF modeling reconstruction (True X) on Siemens Biograph TruePoint TrueV PET/CT. Ann. Nucl. Med..

[32] Jijun L (2010). Abnormal expression of dopamine and serotonin transporters associated with the pathophysiologic mechanism of Tourette syndrome. Neurol. India.

[33] Kugaya A (2003). Changes in human *in vivo* serotonin and dopamine transporter availabilities during chronic antidepressant administration. Neuropsychopharmacol. Off. Publ. Am. Coll. Neuropsychopharmacol..

[34] SCAHILL L (1997). Fluoxetine Has No Marked Effect on Tic Symptoms in Patients with Tourette’s Syndrome: A Double-Blind Placebo-Controlled Study. J. Child Adolesc. Psychopharmacol..

[35] Frokjaer VG (2008). Evaluation of the Serotonin Transporter Ligand 123I-ADAM for SPECT Studies on Humans. J. Nucl. Med..

[36] Knoll P (2012). Comparison of advanced iterative reconstruction methods for SPECT/CT. Z. Med. Phys..

[37] Harris GJ (1992). Putamen volume reduction on magnetic resonance imaging exceeds caudate changes in mild Huntington’s disease. Ann. Neurol..

[38] Koehler PR (1985). MR measurement of normal and pathologic brainstem diameters. Am. J. Neuroradiol..

[39] Mol Debes NMM, Hjalgrim H, Skov L (2008). Limited knowledge of Tourette syndrome causes delay in diagnosis. Neuropediatrics.

[40] Knoop BO, Geworski L, Hofmann M, Munz DL, Knapp WH (2002). Use of recovery coefficient as a test of system linearity of response in positron emission tomography (PET). Physics in Medicine and Biology.

[41] Catafau AM (2004). Impact of dopamine transporter SPECT using123I-Ioflupane on diagnosis and management of patients with clinically uncertain parkinsonian syndromes. Mov. Disord..

[42] Catafau AM, Pérez V, Penengo MM, Al. E (2005). SPECT of Serotonin Transporters Using 123 I-ADAM: Optimal Imaging Time After Bolus Injection and Long-Term Test – Retest in Healthy Volunteers. J Nucl Med.

[43] Kuikka JT (2004). Quantitative accuracy of serotonergic neurotransmission imaging with high-resolution 123I SPECT. NuklearMedizin.

[44] LECKMAN JF (1989). The Yale Global Tic Severity Scale: Initial Testing of a Clinician-Rated Scale of Tic Severity. J. Am. Acad. Child Adolesc. Psychiatry.

[45] Goodman WK (1989). The Yale-Brown Obsessive Compulsive Scale: I. Development, Use, and Reliability. Arch. Gen. Psychiatry.

[46] Beck AT, Ward CH, Mendelson M, Mock J, Erbaugh J (1961). An Inventory for Measuring Depression. Arch. Gen. Psychiatry.

[47] Laux, L., Glanzmann, P., Schaffner, P. & Spielberger, C. Das State-Trait-Angstinventar: STAI. *Weinheim: Beltz* (1981).

[48] Christiansen H (2011). German validation of the Conners Adult ADHD Rating Scales-self-report (CAARS-S) I: Factor structure and normative data. Eur. Psychiatry.

[49] Retz-Junginger P (2002). Wender Utah rating scale. The short-version for the assessment of the attention-deficit hyperactivity disorder in adults. Nervenarzt.

[50] Derogatis LR, Lipman RS, Covi L (1973). The SCL-90: An outpatient psychiatric rating scale. Psychopharmacol. Bull..

[51] Lehrl S, Triebig G, Fischer B (1995). Multiple choice vocabulary test MWT as a valid and short test to estimate premorbid intelligence. Acta Neurol. Scand..

