# Author Correction: Serotonin transporter binding is increased in Tourette syndrome with Obsessive Compulsive Disorder

**DOI:** 10.1038/s41598-020-68278-7

**Published:** 2020-07-01

**Authors:** K. R. Müller-Vahl, N. Szejko, F. Wilke, E. Jakubovski, L. Geworski, F. Bengel, G. Berding

**Affiliations:** 1 Clinic of Psychiatry, Socialpsychiatry and Psychotherapy, Hannover Medical School, Hannover, Germany; 2 Department of Neurology, Medical University of Warsaw, Warsaw, Poland; 3 Department of Bioethics, Medical University of Warsaw, Warsaw, Poland; 4 Department of Medical Physics and Radiation Protection, Hannover, Germany; 5 Department of Nuclear Medicine, Hannover Medical School, Hannover, Germany

Correction to: *Scientific Reports* https://doi.org/10.1038/s41598-018-37710-4, published online 30 January 2019

This Article contains an error in the title of Table 3.

“SERT occupancy (= percentage of decrease in specific over non-specific binding due to treatment) in different brain regions after treatment with escitalopram in patients with TS + OCD and pure OCD (n = 13) depending on quantification procedure.”

should read:

“SERT occupancy (= percentage of decrease in specific over non-specific binding due to treatment) in different brain regions after treatment with escitalopram in patients with TS + OCD (n = 8) depending on quantification procedure.”

In addition, the following text in the Results section contains typographical errors:

“Treatment with escitalopram in patients with TS + OCD and pure OCD (n = 13) resulted in a significant reduction of SERT binding in all investigated brain areas including caudate, putamen, thalamus, hypothalamus, midbrain, pons, and mesial temporal cortex (*p* values between 0.0409 and < 0.0001 depending on the brain region) with differences ranging from 19 to 78% (for further details see Table 3, and Figs. 1, 2). Thus, treatment with escitalopram resulted in a much larger difference in SERT binding compared to the differences detected between untreated patients (with TS + OCD and OCD) and healthy controls at baseline (range 8–11%, for further details see Table 2).”

should read:

“Treatment with escitalopram in patients with TS + OCD (n = 8) resulted in a significant reduction of SERT binding in all investigated brain areas including caudate, putamen, thalamus, hypothalamus, midbrain, pons, and mesial temporal cortex (*p* values between 0.0409 and < 0.0001 depending on the brain region) with differences ranging from 19 to 79% (for further details see Table 3, and Figs. 1, 2). Thus, treatment with escitalopram resulted in a much larger difference in SERT binding compared to the differences detected between untreated patients (with TS + OCD) and healthy controls at baseline (range 8–11%, for further details see Table 2).”

